# The novel anti-colitic effect of β-adrenergic receptors *via* modulation of PS1/BACE-1/Aβ axis and NOTCH signaling in an ulcerative colitis model

**DOI:** 10.3389/fphar.2022.1008085

**Published:** 2022-10-25

**Authors:** Salma Nasser, Dalaal M. Abdallah, Kawkab A. Ahmed, Yousra Abdel-Mottaleb, Hanan S. El-Abhar

**Affiliations:** ^1^ Pharmacology, Toxicology and Biochemistry Department, Faculty of Pharmacy, Future University in Egypt (FUE), New Cairo, Egypt; ^2^ Pharmacology and Toxicology Department, Faculty of Pharmacy, Cairo University, Cairo, Egypt; ^3^ Pathology Department, Faculty of Veterinary Medicine, Cairo University, Giza, Egypt

**Keywords:** mirabegron, carvedilol, presenilin, PPAR-γ, notch, β-Amyloid, smad-7, adherens junction

## Abstract

Although dysautonomia was documented in inflammatory bowel disease, with activation of the stress-related sympathetic system, the role of agonists/antagonists of the adrenergic receptors is not conclusive. Moreover, ulcerative colitis was recently linked to dementia, but the potential role of the presenilin 1(PS1)/BACE-1/beta-amyloid (Aβ) axis has not been evaluated. Hence, we investigated the impact of mirabegron (β3-agonist) and/or carvedilol (β1/β2 antagonist) on iodoacetamide-induced ulcerative colitis with emphasis on the novel pathomechanism of the PS1/BACE-1/Aβ axis in ulcerative colitis, and its relation to the inflammatory cascade, fibrotic processes, and the gut barrier dysfunction. Ulcerated rats were either left untreated or treated for 8 days with mirabegron and/or carvedilol. Besides minimizing colon edema and weight loss, and improving colon structure, mirabegron and/or carvedilol abated colonic PS1/BACE-1/Aβ axis and the NOTCH1/NICD/HES1 hub besides the inflammatory cascade GSK3-β/NF-κΒ/TNF-α, and the oxidative stress marker malondialdehyde. The anti-fibrotic effect was verified by boosting SMAD-7 and inhibiting TGF-β1, α-SMA immunoexpression, and MTC staining. Moreover, the drugs improved the gut barrier function, attested by the increased goblet cells and expression of E-cadherin, and the inhibited expression of *p* ^(Y654)^-β-catenin to preserve the E-cadherin/β-catenin adherens junction (AJ). These signaling pathways may be orchestrated by the replenished PPAR-γ, a transcription factor known for its anti-colitic effect.

**Conclusion:** Besides maintaining the gut barrier, mirabegron and/or carvedilol mediated their anti-colitic effect by their anti-oxidant, anti-inflammatory, and anti-fibrotic capacities. The therapeutic effect of these drugs depends partly on suppressing the harmful signaling pathways PS1/BACE-1/Aβ, NOTCH1/NICD/HES1, GSK3-β/NF-κΒ/TNF-α, and TGF-1β/α-SMA while enhancing PPAR-γ, SMAD-7, mucus, and AJ.

## Graphical Abstract

Mirabegron and carvedilol anti-colitic effect. Both act by inhibiting PS1/BACE-1/Aβ, NOTCH1/NICD/HES1, GSK-3β/NF-κB/TNF-α, MDA, & TGF-β1/α-SMA, while boosting PPAR-γ, SMAD-7 to reduce amyloidosis, osidative stress, inflammation, and fibrosis, while amending E-cadherin/β-catenin adherens junction to enhance gut barrier integrity and replenishing goblet cells to protect the colon.
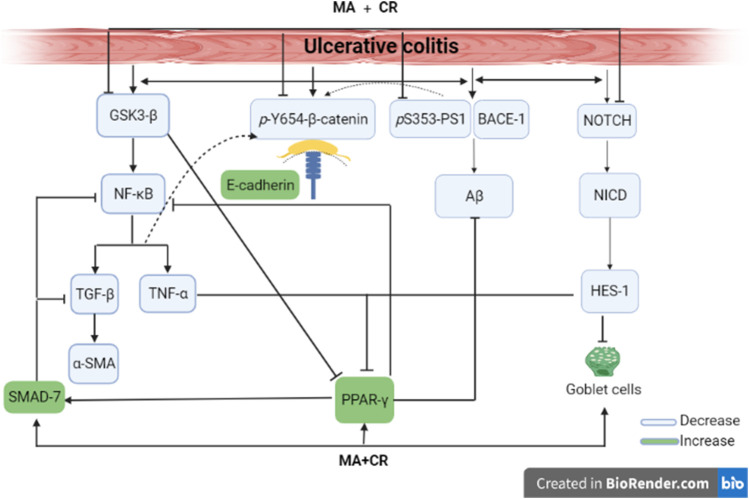

## Introduction

Ulcerative colitis is an inflammatory disease known to show a relapsing-remitting course pattern. The disease is highly prevalent affecting around 200–250 individuals per 100,000 and its widespread is not limited to gender, age, or ethnicity (Kobayashi et al., 2020; Bagalagel et al., 2022). Ulcerative colitis is caused by multiple factors that collectively compromise gut integrity, including genetic susceptibility, environmental factors, and immune dysregulation (Choi et al., 2017). The jeopardization of the intestinal barrier plays a crucial role in the pathophysiology of ulcerative colitis; this can be driven by epithelial cell dysfunction (Gitter et al., 2001; Mehta et al., 2015) or an inflammatory storm that compromises cells in the lamina propria, which leads to chronicity (Strober and Fuss, 2011). Moreover, colon fibrosis can ensue upon exposure to prolonged inflammation (Wynn, 2008; Binabaj et al., 2019).

Despite the extensively studied pathogenesis of this disease, the full mechanism responsible has not yet been fully elucidated. For instance, the possible deposition of amyloid and its role in the pathogenesis of this disease has not been extensively studied. For instance, the intestine of patients with Alzheimer’s disease (AD) revealed the expression of amyloid precursor protein (APP) (Shankle et al., 1993), and a plaque-like deposition (Arai et al., 1991) in the enteric nervous system (ENS). Additionally, it was reported that transgenic mice expressing mutant presenilin-1 (PS1), a critical component for the γ-secretase complex, or its precursor APP had a progressive accumulation of amyloid beta (Aβ) in the ENS (Puig et al., 2015). Indeed, the phosphorylation of PS1 causes a pathogenic conformational change in PS1/γ-secretase that increases the Aβ42/40 ratio (Maesako et al., 2017). These depositions decrease the number of enteric neurons and increase vulnerability to intestinal inflammation. Recent evidence also indicates that irritable bowel disease (IBD) has a positive correlation with the development of dementia (Zhang et al., 2021) and more specifically, the elevated proinflammatory cytokines and the increase in oxidative stress, cause blood-brain barrier dysfunction and accelerate the amyloid-β accumulation in the brain (Kesika et al., 2021). This notion is strengthened by evidence from studies of ADLP^APT^ mice, which carry three human transgenes: APP, PS1, and tau. These mice were found to have altered gut microbiota composition, as well as gut barrier dysfunction and chronic gut and systemic inflammation (El-Hakim et al., 2022).

Apart from its role in the cleavage of APP, PS1 also aids in the cleavage of NOTCH (De Strooper and Annaert, 2001). The impact of the NOTCH signaling pathway on ulcerative colitis has attracted more attention in the recent past (Lin et al., 2019; Li et al., 2020; Christopoulos et al., 2021; Roy et al., 2021). Previously, the overexpression of the NOTCH1 gene was recorded in the colon of dextran sulfate sodium-induced colitic mice (Noah and Shroyer, 2013) and its downstream target gene hairy and enhancer of split 1 (HES1) was reported to deplete the mucus-producing goblet cells (GCs) (Zheng et al., 2011). Indeed, this hub is a dominant regulator of cell fate during intestinal homeostasis, which indicates its crucial role in the pathogenesis of intestinal diseases partly by activating the inflammatory transcription factor nuclear factor kappa Β (NF-κB) (Zhang et al., 2010) and the pro-inflammatory surrogate marker tumor necrosis factor (TNF-α) (Ghorbaninejad et al., 2019). One of the upstream molecules of NF-κB is the glycogen synthase kinase-3 (GSK-3) β, a serine-threonine protein kinase that plays a pivotal role in ulcerative colitis. Aside from its positive correlation with PS1 and Aβ42 (Zhang et al., 2020), previous studies have reported the abundance of this kinase in an ulcerative colitis model (Uddin et al., 2013; Di Gregorio et al., 2017). Furthermore, its inhibition by selective inhibitors has been found to inhibit inflammation and tissue injury in acutely colitic rats by causing downregulation of NF-κΒ (Tantray et al., 2017).

In addition, GSK-3β can regulate peroxisome-proliferator activated receptor gamma (PPAR-γ), another transcription factor that plays an important role in anti-inflammatory reactions possibly by inhibiting the release of TNF-α (Xu et al., 2017). Moreover, in different inflammatory disease conditions, its agonists have been shown to offer protective effects *via* downregulating pro-inflammatory cytokines and inhibiting apoptosis (Vetuschi et al., 2018). It has also been recounted that the anti-inflammatory activity of 5 amino-salicylic acid (5-ASA), a first-line medication for the treatment of ulcerative colitis, requires the activation of epithelial PPAR-γ signaling, which makes it a potential target for therapeutic intervention in ulcerative colitis (Cevallos et al., 2021).

Regarding ulcerative colitis disease, no definitive treatments are present, which makes it attractive to repurpose available molecules. Despite the scarce data available on the role of adrenergic receptors (ARs) in this ailment, some studies correlate between gastrointestinal tract (GIT) and these ARs. Although in IBD patients the balance of autonomic nervous function is disturbed with sympathetic over-activity (Bai et al., 2009), the role of β-AR antagonism in protecting against ulcerative colitis is not conclusive. Previous studies reported that acetic acid-induced ulcerative colitis was alleviated using carvedilol, a β-and α1- AR blocker (Fatani et al., 2015), and nebivolol, a highly selective β1-AR antagonist with a weak β2-AR antagonistic effect and a β3-AR agonism (Aly Labib et al., 2016). These treatments were thought to be effective in their anti-inflammatory, antioxidant, and/or antiapoptotic properties. On the other hand, trinitrobenzene sulfonic acid (TNBS)-induced colitis was found to reduce the expression of β2-AR (Fan et al., 2013), and the administration of salbutamol, a β2-AR agonist, has also been found to ameliorate ulcerative colitis (Deng et al., 2022)**.**

Concerning β3-AR, earlier studies reported that agonists of this receptor possess anti-motility effects on the colon (Lipworth, 1996). This was thought to occur by inhibiting pacemaker potentials (Wu et al., 2015), which suggests a possible role in the treatment of irritable bowel syndrome. Moreover, another study recounted that colitis-induced loss of the β3-AR is one reason behind diarrhea associated with this disease (Zhao et al., 2001). Moreover, the selective β3-agonist, SR58611, was found to reduce the severity of colitis *via* its anti-inflammatory potential and the upregulation of the β3 AR expression in inflamed tissues (Vasina et al., 2008). These findings were supported by the presence of the β3-AR on myenteric neurons to suggest a potential therapeutic target for gut inflammatory disease. The first β3-AR agonist is mirabegron (Bragg et al., 2014), which besides its approved use in the treatment of overactive urinary bladder syndrome (Mo et al., 2017), has other off-target effects by acting on muscarinic receptors, α1-AR, dopamine/noradrenaline transporters, and the sodium channel site 2 (Dehvari et al., 2018).

Based on the role of the dysregulated ARs in IBD (Jacobson et al., 2021), we aimed at studying the potential anti-colitic efficacy of mirabegron and/or carvedilol against the iodoacetamide (IAA)-induced ulcerative colitis model in rats. Our analyses emphasized the colonic PS1/BACE-1/Aβ hub, the NOTCH1 axis, physical barrier dysfunction, PPAR-γ/NF-κB, and the associated fibrotic process.

## Material and methods

### Animals

Adult male Wistar rats (180–250 g) were housed under standard conditions (i.e., at 25°C ± 2°C, humidity 60%–70%, 12 h dark/light cycles) with free access to food and water. Before the experiment, animals were acclimated in cages for 1 week at the Future University in Egypt (FUE; Cairo, Egypt). The study protocol was approved by the Ethics Committee of the Faculty of Pharmacy, Cairo University (Cairo, Egypt) (PT 2288, 25/3/2019) and complies with the National Institutes of Health Guide for the care and use of laboratory animals (NIH, revised 2011). All experiments comply with ARRIVE guidelines.

### Induction of colitis

Rats were fasted for 24 h with free access to water then anesthetized using thiopental (30 mg/kg; i.p). A single dose of 0.15 ml of 4% IAA (Sigma-Aldrich, MO, USA; in 1% methylcellulose) was instilled rectally *via* a catheter placed 8 cm proximal to the anus (Satoh et al., 1997) and animals were kept upside-down to prevent leakage of solution. Animals were then left for 7 days.

### Experimental design

Animals were allocated into 6 groups (n = 6 each) comprising normal and ulcerated rats. Animals in the first group served as the normal control and rats in group 2 were assigned as the untreated ulcerated group. Rats in both groups were administered saline orally for 7 days. Ulcerated animals in groups 3 and 4 received carvedilol (CR; Selleckchem, TX, USA) at doses of 15 and 30 mg/kg (Fatani et al., 2015), whereas rats in group 5 were treated with mirabegron (MA; 30 mg/kg; Selleckchem, TX, USA) based on a pilot study testing 3 (Hatanaka et al., 2013), 10 (Sawada et al., 2013), and 30 mg/kg (de Oliveira et al., 2019; Supplementary Figures S1, S2). In the last group, ulcerated rats received a combination of mirabegron with the low dose of carvedilol (MA+CR15). All treatments were gavaged orally for 7 days.

### Sampling and assessment of body weight changes and colon edema index

Rats were weighed before induction of colitis and on the day of scarification to calculate the difference in body weight. The colon edema index was calculated according to the following formula (Ahmedy et al., 2020):

Colon edema index=weight of the colon (mg)/final body weight (g)

Following, the distal 10 cm of the colon was opened longitudinally and divided into two halves; the first half (n = 3) was fixed in 10% neutral buffered formalin for histopathological/immunological examinations, whereas the 2nd was further subdivided into 2 parts. The first portion (n = 6) was homogenized in normal saline for biochemical and ELISA assessments and the 2nd portion (n = 4) was kept in RIPA buffer with a protease inhibitor to assess the Western blot parameters. All samples were divided into aliquots and stored at −80°C.

### Measurement of colonic malondialdehyde content

The lipid peroxide content was measured colorimetrically at a wavelength of 534 nm using an MDA kit (Bio-diagnostic, Cairo, Egypt; Cat #: MD2529) and processed according to the manufacturer’s instructions.

### ELISA technique

The colon contents of NOTCH1 (MyBioSource, CA, USA, Cat#: MBS004226), E-cadherin (MyBioSource, Cat#: MBS761750), Aβ_(1–42)_ peptide (MyBioSource, Cat#: MBS726579), SMAD7 (MyBioSource, Cat#: MBS2501417), PPAR-γ (MyBioSource, Cat#: MBS2508012), TNF-α (CUSABIO, TX, USA; Cat#: CSB-E11987r), and HES1 (LifeSpan Biosciences, WA, USA; Cat #: LS-F33052) were assessed using rat ELISA kits according to the manufacturer’s directions, and the produced color was measured spectrophotometrically at a wavelength 450 nm.

### Western blot analysis

The protein concentration in the lysed samples was determined using the Bradford Protein Assay kit (BIO BASIC, ON, Canada, Cat# SK3041) according to the manufacturer’s instructions. Equal sample protein concentrations were added to 2x Laemmli sample buffer and heated at 95°C for 5 min, then loaded on the SDS-PAGE gel. The blots were transferred to a nitrocellulose membrane that was blocked with TEST buffer and 3% BSA at room temperature for 1 h, then incubated overnight at 4°C with the primary antibodies (ThermoFisher Scientific, MA, USA) for *p* ^(S353)^-PS1 (Cat #: PA5-64705), Total-PSI (Cat #: PA5-96088), phosphorylated glycogen synthase kinase (*p* ^(S9)^ -GSK3β; Cat #: MA5-14873), *p* ^(Y654)^-β-catenin (Cat #: PA5-36748), BACE-1 (Cat #: PA1-757), NICD (Cat #: MA5-11961), or β-actin (Cat #: AM4302) for protein normalization diluted in TEST. The blots were then rinsed 5 times with TEST each 5 min then incubated with the horseradish peroxidase (HRP)-conjugated 2^ry^ antibody for 1 h at room temperature, and the blots were rinsed again. The chemiluminescent substrate (Clarity™ Western ECL substrate-Bio-Rad, CA, USA; Cat#170–5,060) was applied to the blot according to the manufacturer’s recommendation and the signals of target protein bands were captured using a CCD camera-based imager and analyzed using the Chemi Doc MP Bio-Rad imager (CA, USA #170–5,060) software.

### Histopathological H&E alcian blue, and masson’s trichome staining

The fixed colon specimen was processed in paraffin blocks by regular method and sectioned at 4 μm thickness and stained by H&E then examined by a BX43 light microscope (Olympus; Tokyo, Japan) in a blinded fashion. Quantitative histological assessment of colon lesions was carried out and scored from (0 – 3) in five randomly examined microscopic fields per animal as follows: (0) no changes, 1) mild, 2) moderate, and 3) severe changes for mucosal necrosis, inflammatory cells infiltration, edema, hemorrhage, fibrosis, and depletion of mucous secreting glands (Riley et al., 1988; Salem and Wadie, 2017). The deparaffinized blocks were stained with Alcian blue for the determination of goblet cells (GCs) and Masson’s Trichrome (MTC) for collagen fibers (Bancroft J. and Layton C., 2019). The area percentage of fibrosis, as well as the number of positive GCs, were blindly visualized and quantified using CellSens dimensions (Olympus Software).

### Immunoreactivity of NF-κB, TGF-β1, and α-SMA

The deparaffinized colon slices were immunostained with primary antibodies (ThermoFisher Scientific) of NF-κB (Cat # 14–6,731-81), TGF-β1 (Cat # MA5-15065), or α-SMA (Cat #: 14–9,760-82), then incubated with goat anti-rabbit IgG (H+L) Superclonal™ Recombinant Secondary Antibody, HRP (Cat # A27036). Positive immune expression was expressed as area % from 5 randomly chosen fields per section using Image J Software 1.46a (NIH, MD, USA).

### Coefficient drug interaction

The equation of coefficient drug interaction (CDI)=AB/(A×B) , was used to test the interaction between a single agent on the parameters showing significant differences between the combination group and either treatment alone. Notably, in each parameter, the AB is the ratio of the combination group to the model group and A or B is the ratio of the CR15 or MA group to the model group. Thus, a CDI value less than, equal to, or greater than 1 indicates that the interaction is synergistic, additive, or antagonistic, respectively (Yan et al., 2009).

### Statistical analysis

For the parametric data, values are presented as mean ± SD that were analyzed using one-ANOVA followed by Tukey’s *posthoc* test for multiple comparisons, *p* < 0.05. The non-parametric data are presented as median (min-max) that were analyzed using Kruskal-Wallis followed by Dunn’s *posthoc* test, *p* < 0.05. Graph pad prism^®^ software package, version 7 (CA, USA) was used for calculation and drawing.

## Results

### Mirabegron and/or carvedilol increase body weight and lessen colon edema index in rats with IAA-induced ulcerative colitis

**FIGURE 1:**
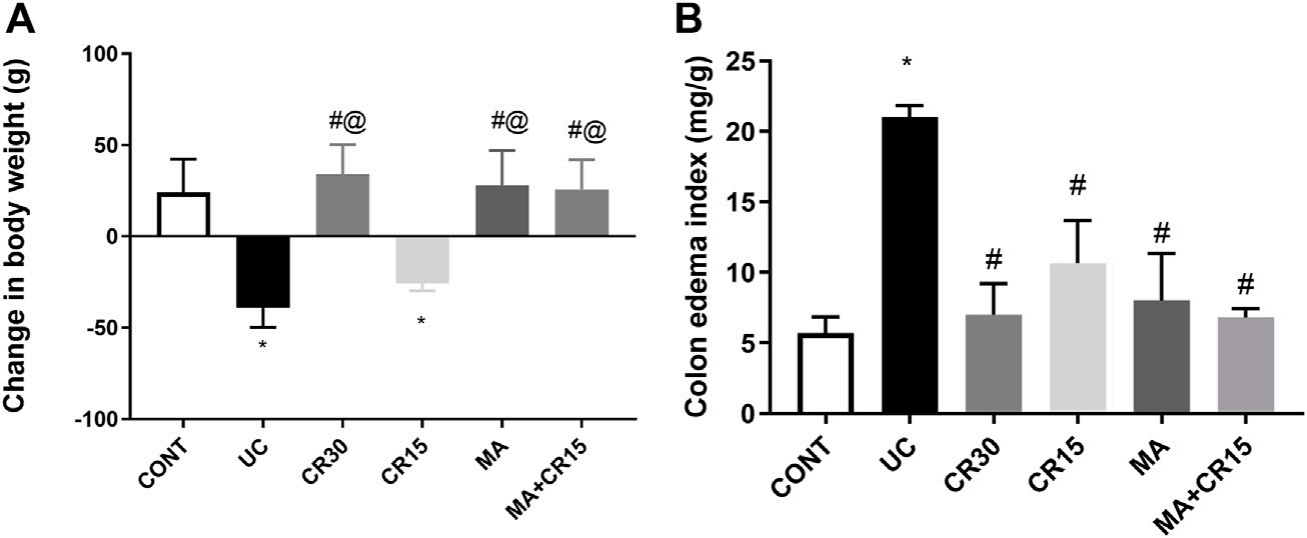
Effect of mirabegron and/or carvedilol on **(A)** body weight, and **(B)** colon edema index in rats with IAA-induced ulcerative colitis. Data are presented as mean ± SD (n = 6 rats/group). Statistical analysis was carried out using one-way ANOVA followed by Tukey’s *posthoc* test; *p* < 0.05, as compared to the (*) CONT, (#) UC, and (@) CR15 groups. CONT, control; CR, carvedilol; IAA, iodoacetamide; MA, mirabegron; UC, ulcerative colitis.

As compared to the control group, Figure 1 depicted a 39 g loss of (A) body weight in ulcerated rats relative to the initial weight along with a 3.7-fold increment in (B) colon weight. Nonetheless, all treatment regimens, except the low dose of carvedilol (CR15), restored body weight as compared to the control group, an effect that was extended to abate the colon index.

### Mirabegron and/or carvedilol improve colon histologic structure in rats with IAA-induced ulcerative colitis

**FIGURE 2:**
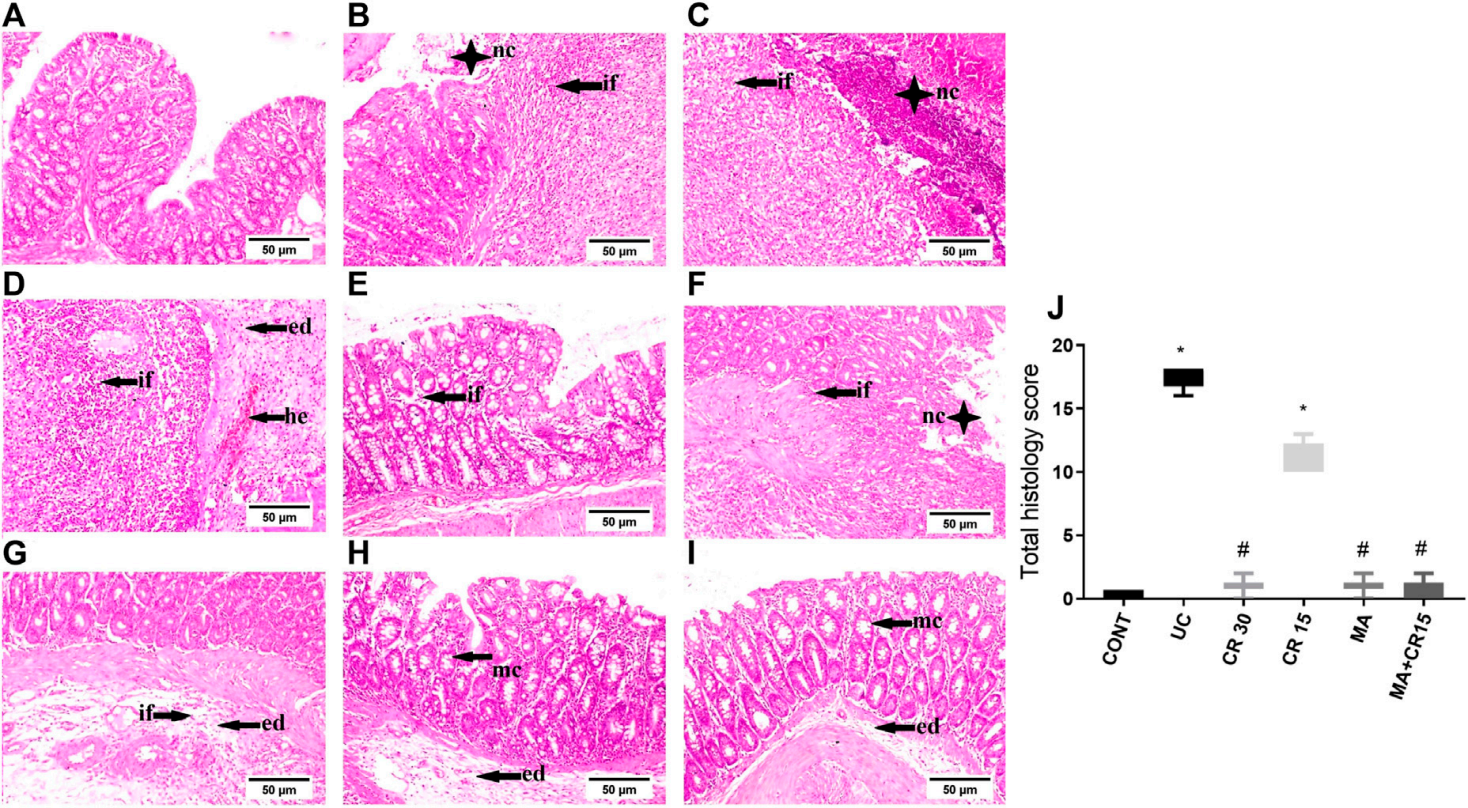
Effect of mirabegron and/or carvedilol on colon histopathological changes and total lesions score in rats with IAA-induced ulcerative colitis. Representative photomicrographs (H&E; scale bar, 50 μm) of the **(B–D)** UC model show marked mucosal necrosis (nc), massive inflammatory cells infiltration (if), marked edema (ed), and focal hemorrhage (he), compared to the section of the **(A)** CONT group that reveals normal morphology. Section of **(E)** CR30 shows few inflammatory cells infiltration in the lamina propria, whereas, sections of **(F,G)** CR15 depict mucosal/submucosal focal necrosis associated with inflammatory cells infiltration, fibroblasts proliferation, and submucosal edema. Treatment with **(H)** MA and **(I)** MA+CR15 reveals hyperplasia of mucous-secreting glands (mc) and slight submucosal edema. **(J)** represents total lesion scoring. Data are presented as median (min-max) (5 fields in each section, n = 3 rats/group); the data were analyzed using Kruskal-Wallis followed by Dunn’s *posthoc* test; *p* < 0.05. As compared to the (*) CONT and (#) ulcerated groups. CONT, control; CR, carvedilol; ed, edema; he, hemorrhage; if, inflammatory cells infiltration; IAA, iodoacetamide; nc, necrosis; MA, mirabegron; mc, mucous secreting glands; UC, ulcerative colitis.

As presented in Figure 2, colon sections of (B-D) ulcerated rats reveal a marked increment in injury markers (mucosal necrosis, inflammatory cells infiltration, edema, hemorrhage, fibrosis, and depletion of mucous secreting glands), as compared to the normal section of the (A) control group. Contrariwise, the section of rats treated with (F) the low dose of carvedilol (CR15) divulges a moderate improvement in the injury markers but a slight upturn in the GCs. Meanwhile, treatment with the (E) high dose of carvedilol (CR30) shows an overall improvement in the colon structure, whereas a better recovery is detected in sections of rats treated with (H) mirabegron and (I) the combination (MA+CR15), with slight hyperplasia of mucous secreting glands and submucosal edema. The collective score of histopathological lesions is shown in panel J.

### Mirabegron and/or carvedilol reduce colon amyloidosis *via* inhibiting PS1, BACE-1, and Aβ_(1–42)_ in rats with IAA-induced ulcerative colitis

**FIGURE 3:**
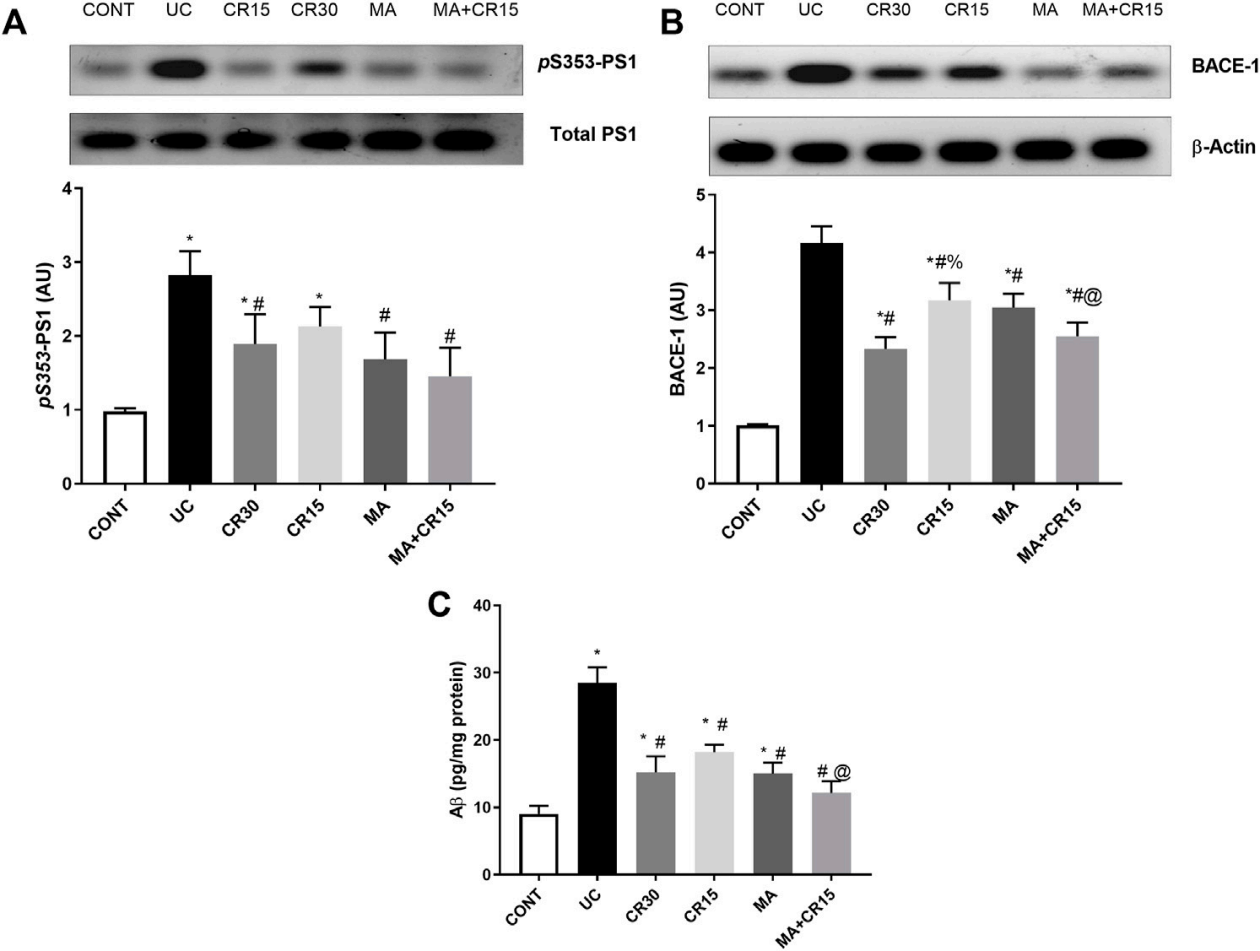
Effect of mirabegron and/or carvedilol on colon protein expression/content of **(A)** *p* ^(S353)^-PS1, **(B)** BACE-1, and **(C)** Aβ_(1–42)_ in rats with IAA-induced ulcerative colitis. Data are presented as mean ± SD (n = 4–6). Statistical analysis was carried out using one-way ANOVA followed by Tukey’s *posthoc* test; *p* < 0.05. As compared to the (*) CONT, (#) UC, and (@) CR15 groups. Aβ, beta-amyloid; BACE-1, beta-site APP-cleaving enzyme; CONT, control; CR, carvedilol; IAA, iodoacetamide; MA, mirabegron; PS1, presenilin; UC, ulcerative colitis.

The IAA-induced colon amyloidosis and the effect of the different treatment regimens were confirmed biochemically (Figures 3A–C). The ulcerated rats showed increments in both the APP degrading enzymes (A) PS1 (2.9 folds) and (B) BACE-1 (4.1 folds) to augment the formation of (C) Aβ (3.2 folds) in the colon, as compared to the control group. Nevertheless, post-treatment with the low dose of carvedilol (CR15) lessened PS1 by only 39%, whereas treatment with the high dose of carvedilol (CR30), mirabegron, and the combination regimen markedly reduced PS1 to reach a non-significant level as compared to the control group. Once more, treatment with MA alone and in combination returned the protein expression of BACE-1 to a comparable level of the control group. However, the effect of high dose of carvedilol (CR30) was less pronounced and the least effect was mediated by the low dose of carvedilol (CR15). Finally, in a comparable manner, the different treatments leveled off the Aβ content, with a better effect mediated by the combination regimen to surpass that of low dose of carvedilol (CR15) alone.

### Mirabegron and/or carvedilol deactivate the colonic NOTCH1/NICD/HES1 trajectory in rats with IAA-induced ulcerative colitis

**FIGURE 4:**
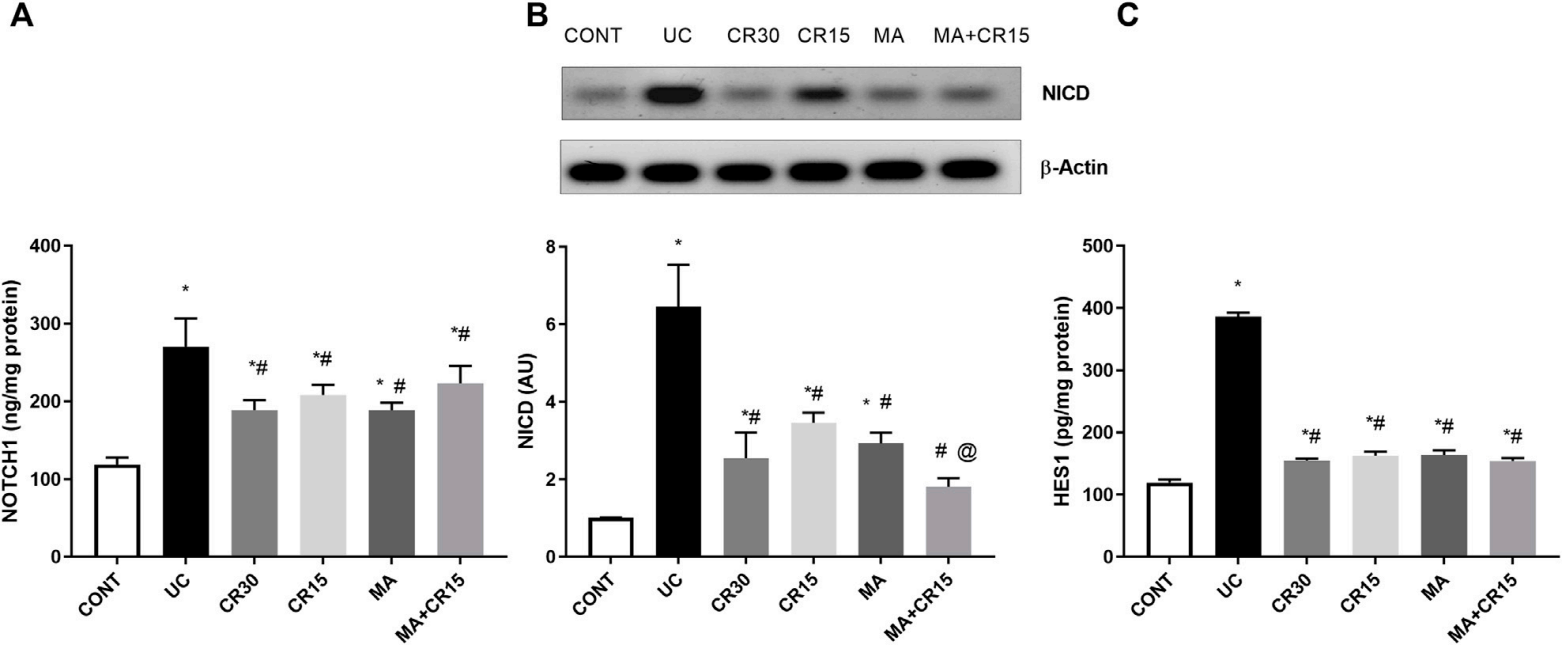
Effect of mirabegron and/or carvedilol on colon protein expression/content of **(A)** NOTCH1, **(B)** NICD, and **(C)** HES1 in rats with IAA-induced ulcerative colitis. Data are presented as mean ± SD (n = 4–6). Statistical analysis was carried out using one-way ANOVA followed by Tukey’s *posthoc* test; *p* < 0.05, as compared to the (*) CONT, (#) UC, and (@) CR15 groups. CONT, control; CR, carvedilol; IA, iodoacetamide; MA, mirabegron; NICD, nuclear intracellular domain; UC, ulcerative colitis.

**FIGURE 5:**
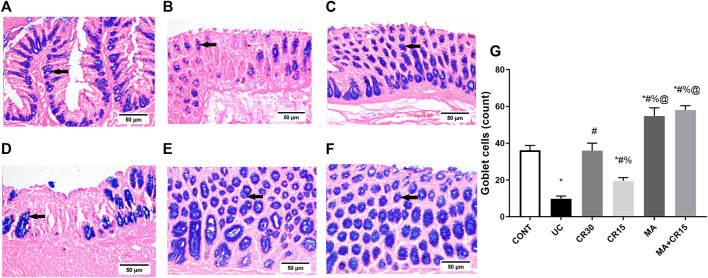
Effect of mirabegron and/or carvedilol on the number of goblet cells in the colon of rats with IAA-induced ulcerative colitis. Data are presented as mean ± SD (n = 3). Statistical analysis was carried out using one-way ANOVA followed by Tukey’s *posthoc* test; *p* < 0.05, as compared to the (*) CONT, (#) UC, (%) CR30, and (@) CR15 groups. Panels **(A–F)** represent colon photomicrographs stained with Alcian blue (scale bar, 50 μm). Section of **(B)** IAA model shows a sharp decrease in the number of GCs, compared to that of **(A)** CONT group. Section of the ulcerated rats treated with **(D)** CR15 shows an increase in GCs, whereas that of the **(C)** CR30 treated group reaches a normal level. However, treatment with **(E)** MA and **(F)** MA+CR15 shows a marked increase in the number of GCs that exceeded that in panel **(A)** (arrows). Panel **(G)** summarizes the above results (5 fields in each section). CONT, control; CR, carvedilol; IAA, iodoacetamide; MA, mirabegron; UC, ulcerative colitis.

The enhanced PS1 also cleaves NOTCH1 to activate its downstream pathway, a verity that was depicted in Figure 4 **,** where IAA boosted the NOTCH1/NICD/HES1 signaling pathway to reach 2.3 (NOTCH1), 6.4 (NICD), and 3.2 (HES1) folds, respectively relative to the normal counterparts. These increments were almost equally reduced by all single regimens to achieve a significant level as compared to either ulcerated or control groups. However, treatment with the combination regimen reduced NICD to a non-significant level relative to the control group, an effect that was better than that of the low dose of carvedilol (CR15) alone. The impact of HES-1 on GC count was also evident from the results of our Alcian blue stained GC count (Figure 5), which revealed a sharp GC reduction in (B) colitic rats relative to the (A) control group. We also observed a strong increase in GC count in the (E) mirabegron-treated group, which exceeded that of the control group, and an even stronger effect in the group receiving the (F) combination regimen. Panel G summarizes these data.

### Mirabegron and/or carvedilol activate colonic PPAR-γ but inhibit the GSK3-β/NF-κB/TNF-α axis and lipid peroxidation in rats with IAA-induced ulcerative colitis

**FIGURE 6:**
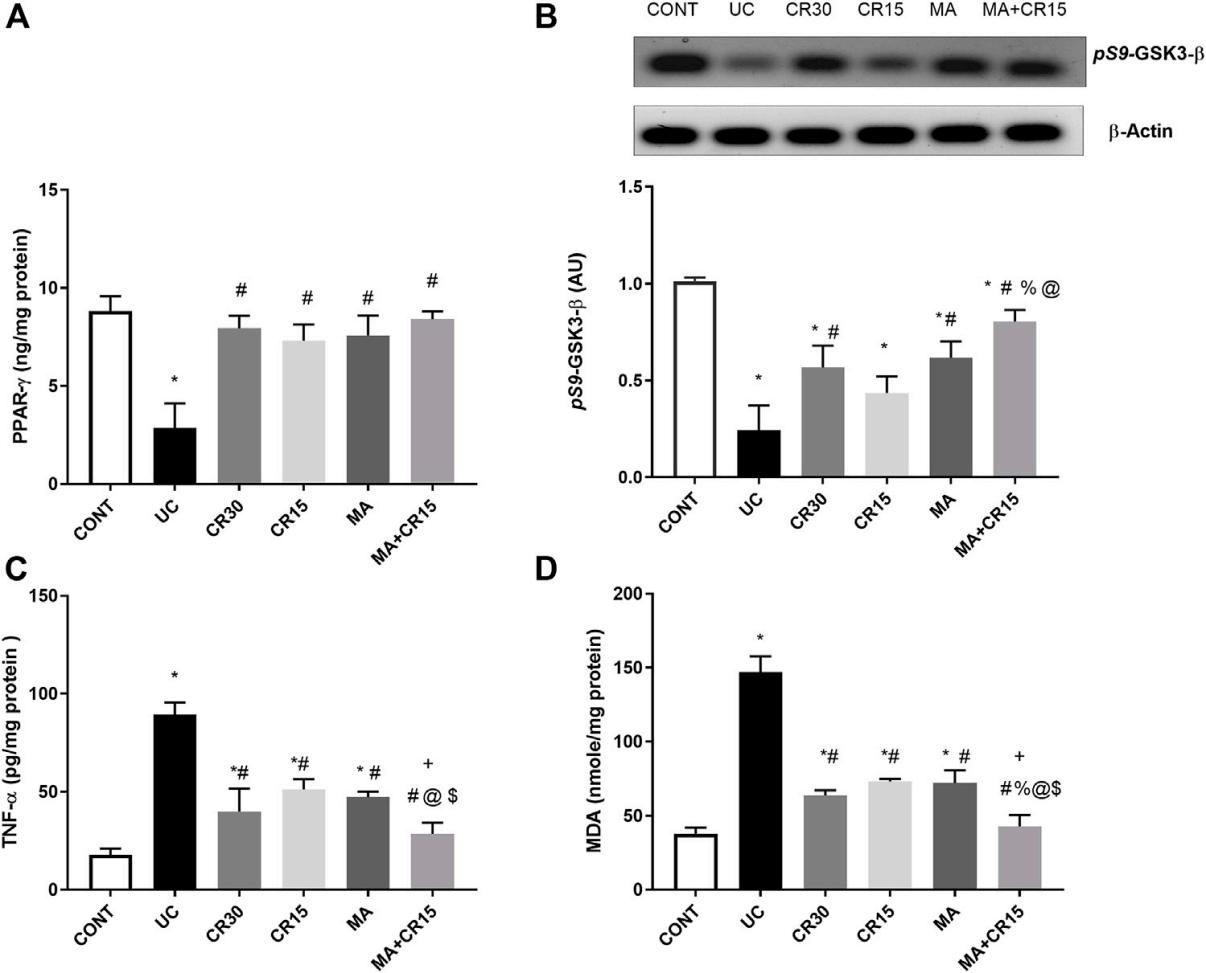
Effect of mirabegron and/or carvedilol on colon protein expression/content of **(A)** PPAR-γ, **(B)** *p* ^(S9)^-GSK3-β, **(C)** TNF-α, and **(D)** MDA in rats with IAA-induced ulcerative colitis. Data are presented as mean ± SD (n = 4–6). Statistical analysis was carried out using one-way ANOVA followed by Tukey’s *post-hoc* test; *p* < 0.05. As compared to the (*) CONT, (#) UC, (%) CR30, (@) CR15, and ($) MA groups. (+) indicate additive interaction using CDI. CDI, coefficient of drug interaction; CONT, control; CR, carvedilol; GSK3-β, glycogen synthase kinase 3 beta; IAA, iodoacetamide; MA, mirabegron; MDA, malondialdehyde; PPAR-γ, peroxisome proliferator-activated receptor gamma; TNF-α, tumor necrosis factor-alpha; UC, ulcerative colitis.

**FIGURE 7:**
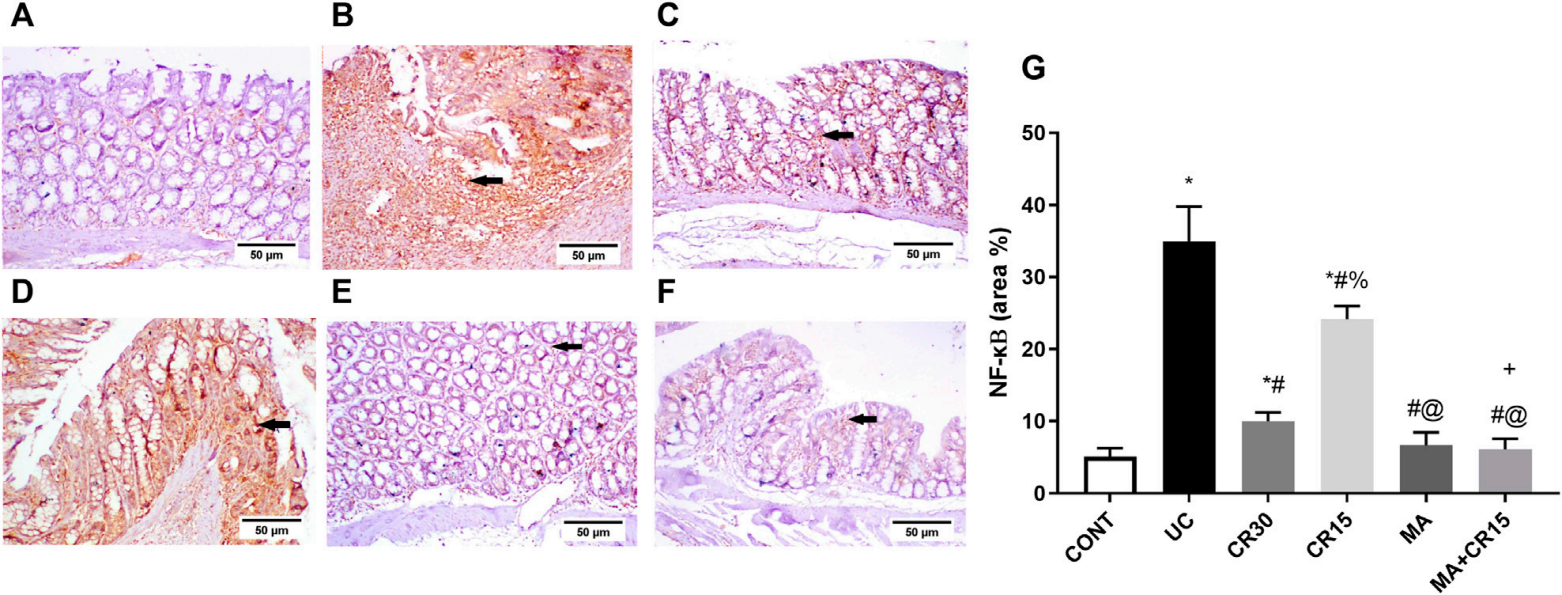
Effect of mirabegron and/or carvedilol on colon NF-κB immunoreactivity in rats with IAA-induced ulcerative colitis. Panels **(A–F)** represent colon photomicrographs of NF-κB immunoreactivity (scale bar, 50 μm). Section of **(B)** UC model shows strong positive immunostained cells, relative to the **(A)** CONT group that shows no immune expression. Treatment with **(D)** CR15 reduced the number of immunostained cells, whereas the section of **(C)** CR30 lessened it markedly. Sections of **(E)** MA and **(F)** MA+CR15 treated groups show a weak immune expression (arrows). **(G)** shows the % area of NF-κB staining (5 fields in each section). Data are presented as mean ± SD (n = 3). Statistical analysis was carried out using one-way ANOVA followed by Tukey’s *posthoc* test; *p* < 0.05. As compared to the (*) CONT, (#) UC, and (@) CR15. (+) additive interaction using CDI. CDI: coefficient of drug interaction; CONT, control; CR, carvedilol; IAA, iodoacetamide; MA, mirabegron; NF-κB, nuclear factor kappa B; UC, ulcerative colitis.

In Figure 6, the insult revealed its inflammatory effect by reducing (A) PPAR-γ (32%) and (B) *p* ^(S9)^-GSK-3β by 76%, whereas it accelerated the pro-inflammatory mediator (C) TNF-α to 5 folds and the lipid peroxidation marker (D) MDA to 3.9 folds when compared to the control group. Relative to the ulcerated group, all post-treatments normalized PPAR-γ and enhanced *p*-GSK3-β indicating its deactivation to different levels. Treatment with the high dose of carvedilol (CR30) or mirabegron alone elevated inactive *p*-GSK-3β significantly, whereas the addition of mirabegron to the low dose of carvedilol (CR15) enhanced its effect. Moreover, all treatments showed a comparable reduction in colon contents of TNF-α and MDA, except for the combination regimen, which normalized these contents and showed an additive interaction between the two drugs. The inflammatory condition was further strengthened by the strong protein expression of the transcription factor NF-κB (Figure 7B), compared to the section of (A) control normal rats. This expression was inhibited significantly with the best effect seen in sections of (E) mirabegron and (F) the combination (MA+CR15) that reveal a weak immune expression to mimic that of panel A. While the worst effect was seen in sections of (D) CR15 and a moderate decrement was seen in section of (C) CR30. These results are summarized in panel G.

### Mirabegron and/or carvedilol modulate the TGF-β1/α-SMA/SMAD-7 hub in rats with IAA-induced ulcerative colitis

**FIGURE 8:**
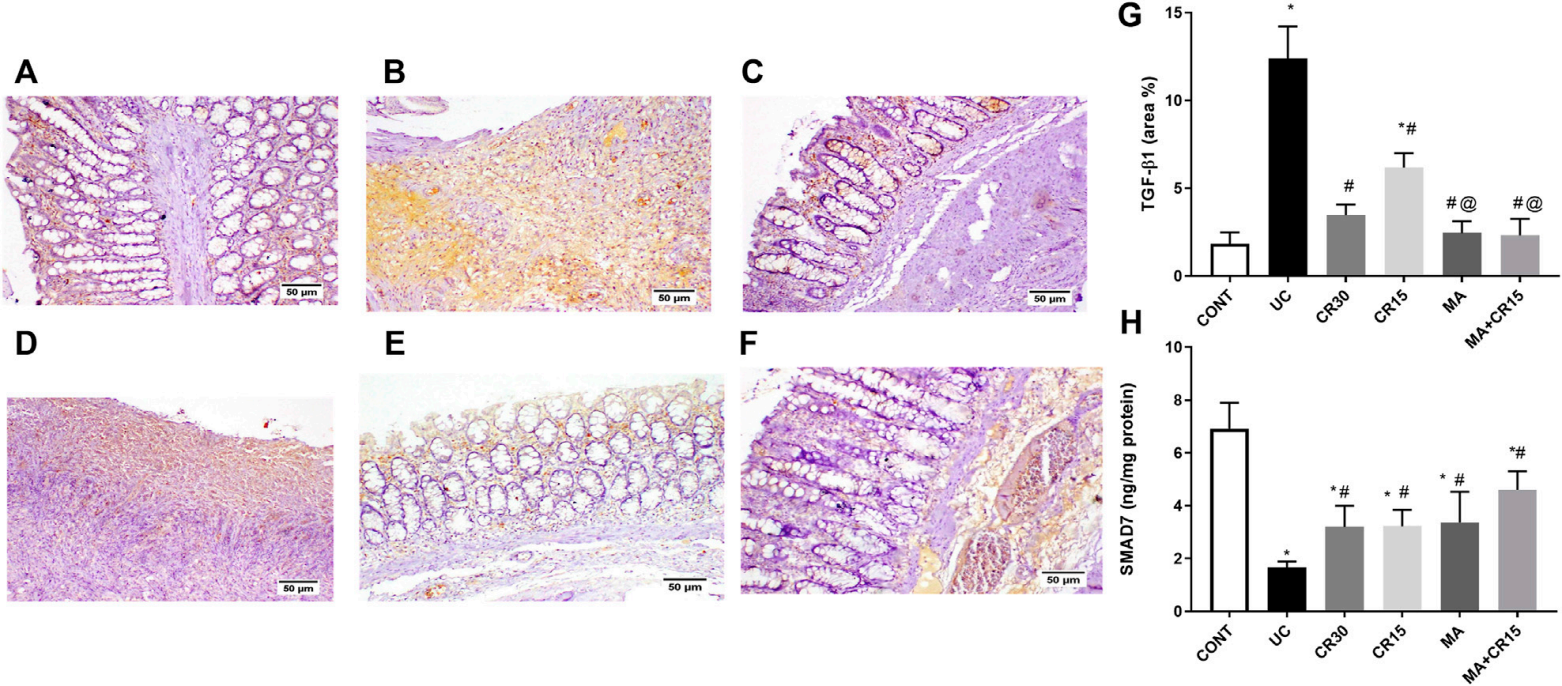
Effect of mirabegron and/or carvedilol on colon TGF-β1 immunoreactivity and SMAD7 content in rats with IAA-induced ulcerative colitis. Section of **(A)** CONT group, shows a normal expression of TGF-β1, while that of **(B)** UC group shows increased expression of TGF-β in the ulcerative mucosa. Treatment with **(C)** CR30 reveals moderate expression of TGF-β1, whereas treatment with **(D)** CR15 only lessened the expression of TGF-β1. Sections of **(E)** MA and **(F)** MA+CR15 treated groups show few expressions of TGF-β1 in the mucosa. **(G)** presents the % area of TGF-β1 staining (5 fields in each section) and **(H)** represents the effect on SMAD-7. Data are presented as mean ± SD (n = 3–6). Statistical analysis was carried out using one-way ANOVA followed by Tukey’s *posthoc* test; *p* < 0.05, as compared to the (*) CONT, (#) UC, and (@) CR15 groups. CONT, control; CR, carvedilol; IAA, iodoacetamide; MA, mirabegron; TGF-β1, transforming growth factor beta; UC, ulcerative colitis.

**FIGURE 9:**
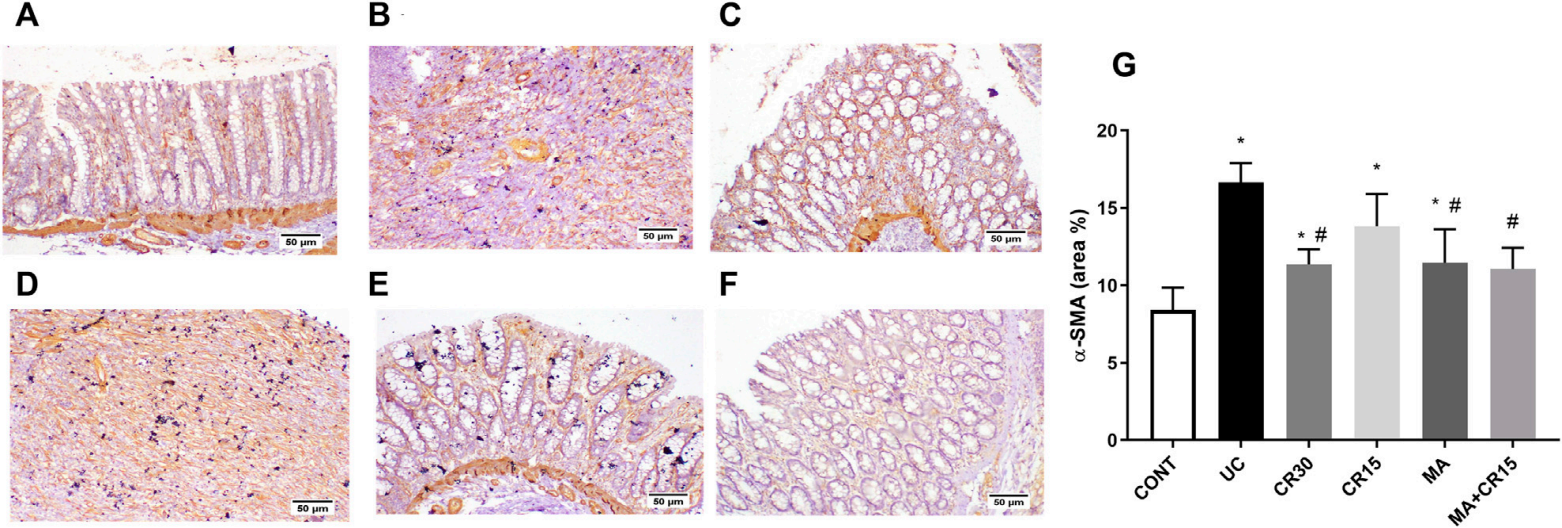
Effect of mirabegron and/or carvedilol on the colonic α-SMA immunoreactivity in rats with IAA-induced ulcerative colitis. Section of **(A)** CONT group shows a normal expression of α-SMA in the lamina epithelia, lamina propria, and muscularis mucosa, while that of **(B)** UC group and **(D)** CR15 treated group show higher expression of α-SMA in the injured intestinal mucosa. Treatment with **(C)** CR30, **(E)** MA, and **(F)** MA+CR15 reveals few expressions of α-SMA in the mucosa. **(G)** shows the % area of α-SMA staining (6 fields in each section). Data are presented as mean ± SD (n = 3 rats/group). Statistical analysis was carried out using one-way ANOVA followed by Tukey’s *posthoc* test; *p* < 0.05, as compared to the (*) CONT and (#) UC groups. CONT, control; CR, carvedilol; IAA, iodoacetamide; MA, mirabegron; α-SMA, alpha-smooth muscle actin; UC, ulcerative colitis.

As depicted in Figure 8 and compared to the photomicrograph of the (A) control group, the (B) IAA model triggers fibrosis manifested by the increased expression of TGF-β1 (3.6 folds), an effect that was minimized after treatment with the (D) low dose of carvedilol (CR15) and reached a normal expression level in the groups treated with the (C) high dose of carvedilol (CR30), (E) mirabegron and (F) the combination (MA+CR15). These results are summarized in panel G. In an opposite pattern, the IAA-induced ulcerative colitis sharply reduced the anti-fibrotic marker (H) SMAD-7 by 76% *vs* the control group. However, all treatments partially increased it with the highest increment noted in the combination regimen (2.8 folds), as compared to the insult. The IAA-induced elevation in TGF-β1 protein expression entailed its downstream target α-SMA (Figure 9), as depicted in panel (B), and compared to the (A) control group to further confirm the fibrotic signal. The best effect was noticed in the (F) combination-treated group, which showed no significant difference when compared to the control group as depicted in panel (G).

### Mirabegron and/or carvedilol lessen collagen deposition in the colon of rats with IAA-induced ulcerative colitis

**FIGURE 10:**
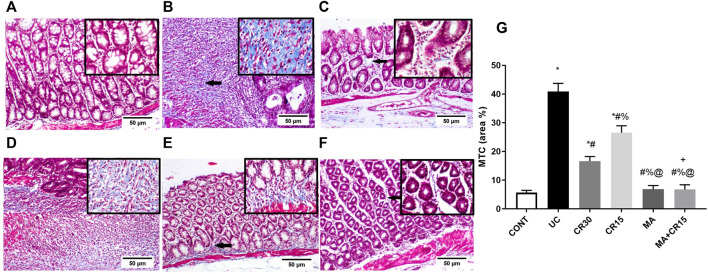
Effect of mirabegron and/or carvedilol on MTC stained collagen fibers in the colon of rats with IAA-induced ulcerative colitis. Panels of A-F represent colon photomicrographs stained with MTC (scale bar, 50 μm). Section of **(A)** CONT shows normal weak MTC stained collagen fibers, whereas the section of **(B)** ulcerated group reveals a marked increase in collagen fibers deposition. Treatment with **(C)** CR30 and **(D)** CR15 show mild and moderate increases in collagen fibers deposition, respectively. However, treatment with **(E)** MA and **(F)** MA+CR15 reveals weak collagen fibers deposition (arrows). **(G)** shows the % area of MTC staining (5 fields in each section). Data are presented as mean ± SD (n = 3 rats/group). Statistical analysis was carried out using one-way ANOVA followed by Tukey’s *posthoc* test; *p* < 0.05, as compared to the (*) CONT, (#) UC, (@) CR15, and (%) CR30 groups. (+) Indicates additive interaction using CDI. CDI, coefficient of drug interaction; CONT, control; CR, carvedilol; IAA, iodoacetamide; MA, mirabegron; MTC, Masson’s Trichrome; UC, ulcerative colitis.

Figure 10 further validates the fibrotic effect of the ulcerative colitis inducer, which causes a marked increase in collagen fibers deposition as shown in section (B), compared to the weak deposition of MTC-stained collagen fibers in the lamina propria of the (A) control group. However, the colon of rats treated with the (D) low dose of carvedilol (CR15) exhibited moderate deposition of collagen fibers to be further reduced in the (C) high dose (CR30)-treated group, but with a slight increase in the lamina propria and submucosa. A near normal deposition was visualized in the colon of rats treated with either (E) mirabegron or (F) the combination (MA+CR15).

### Mirabegron and/or carvedilol augment colon adherens junction in the colon of rats with IAA-induced ulcerative colitis

**FIGURE 11:**
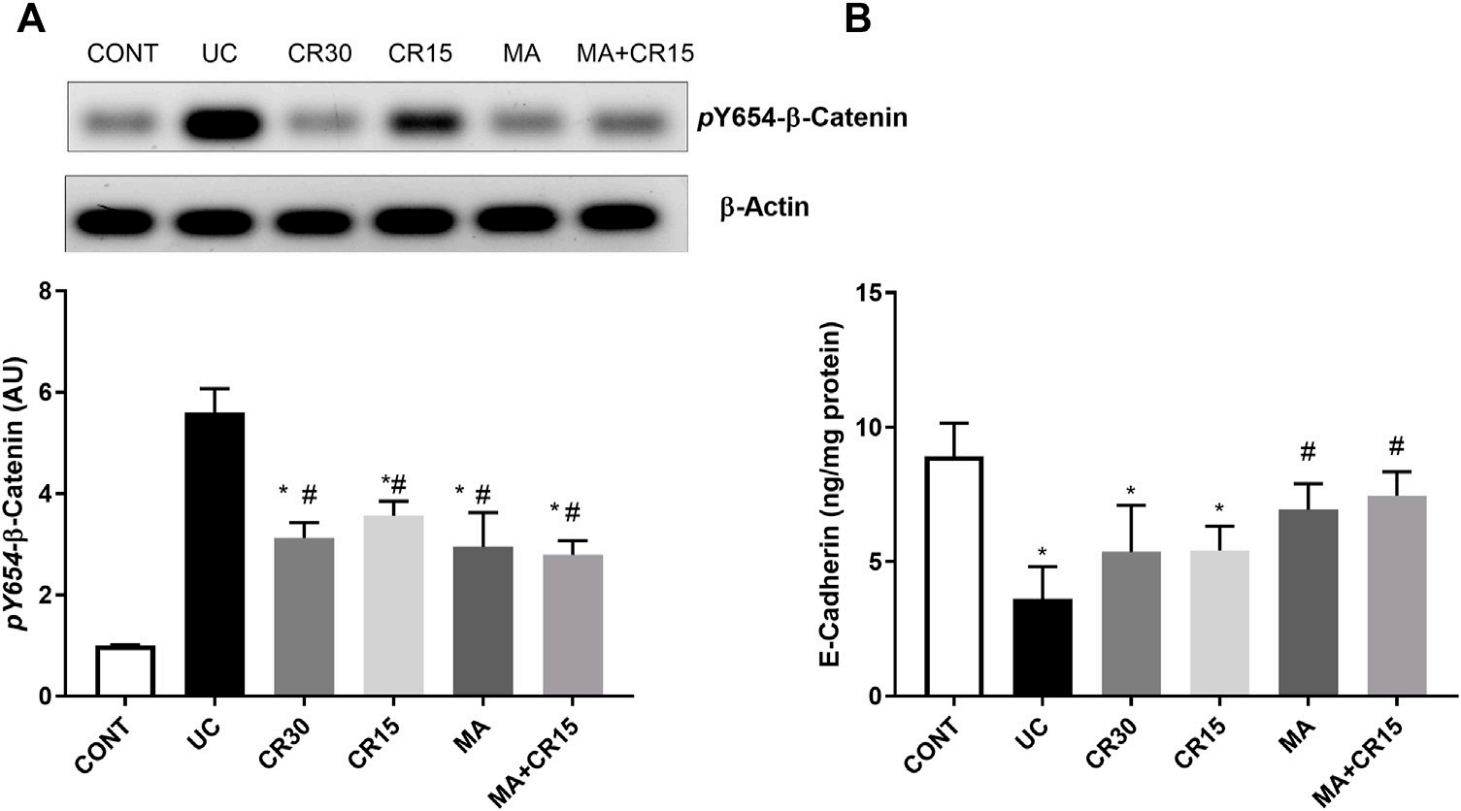
Effect of mirabegron and/or carvedilol on colon protein expression/content of **(A)** *p* ^(Y654)^-β-catenin and **(B)** E-cadherin in rats with IAA-induced ulcerative colitis. Data are presented as mean ± SD (n = 4–6). Statistical analysis was carried out using one-way ANOVA followed by Tukey’s *posthoc* test; *p* < 0.05, as compared to the (*) CONT and (#) UC groups. CONT, control; CR, carvedilol; IAA, iodoacetamide; MA, mirabegron; UC, ulcerative colitis.

Since ulcerative colitis disturbs the membrane integrity, Figure 11 shows the impact of the IAA model on the AJ by studying its effect on the β-catenin/E-cadherin complex. In this figure, IAA caused an upsurge in the protein expression of (A) *p* ^(Y654)^-β-catenin to reach 5.6 folds relative to the control group. On the contrary and to confirm the interrupted colon cell membrane, the insult leveled off the content of (B) E-cadherin by 59%, as compared to the normal rats. However, this picture was inverted in the treated groups, where all treatments decreased the protein expression of *p* ^(Y654)^-β-catenin to a comparable level. Unexpectedly, carvedilol at its two doses failed to affect E-cadherin, while mirabegron alone or in combination succeeded to increase its colon content to reach almost the normal level.

## Discussion

Our study is the first to highlight that the modulation of β-ARs using the β3-AR agonist mirabegron and the β1and β2 antagonist carvedilol offer a therapeutic potential against an ulcerative colitis model. These drugs prove their anti-inflammatory, antioxidant, and anti-fibrotic capacities besides maintaining gut barrier integrity. The anti-colitic mechanisms by which these drugs and their combination act reside partially on shutting down the PS1/BACE-1/Aβ axis, the NOTCH1/NICD/HES1 signaling, and the GSK-3β/NF-κB/TNF-α, as well as the lipid peroxide marker MDA. Besides, they modulate the TGF-β1/α-SMA/ SMAD-7 axis to highlight their anti-fibrotic role. Furthermore, by regulating the β-catenin/E-cadherin hub and the heightening of the GCs these drugs maintain cell integrity. Additionally, the β-AR modulators enhanced PPAR-γ, a transcription factor with an anti-colitic role. Indeed, our results showed that mirabegron has augmented the effect of the low dose of carvedilol to mimic approximately that of the high dose, where the low dose alone exhibited the least beneficial effect.

The impact of the β-ARs on colonic amyloidosis has not been studied before. Indeed, our study is the first to document the presence of amyloidosis-related proteins in a model of ulcerative colitis. Here, the IAA model resulted in an upsurge in the protein expression/content of PS1, BACE-1, and Aβ, whereas post-administration of mirabegron— with or without carvedilol— succeeded to turn this axis off. Amyloidosis is known to accompany AD centrally, but no direct role in ulcerative colitis has yet been reported. Nevertheless, the association between dementia and patients with IBD (Zhang et al., 2021) and the beneficial role of β3 agonism for AD (Tournissac et al., 2021) has been recently highlighted. In addition, earlier studies reported that Aβ or its precursor was present in the enteric neurons of patients with AD (Shankle et al., 1993; Puig et al., 2015; Yi et al., 2017). Although no studies have reported a relation between β3-AR agonists and PS1 or BACE-1, the beneficial role of a β3-AR agonist against Aβ was reported in a model of AD, where it helped to avert the Aβ-induced memory loss, whereas the effect of a β2 agonist was more limited (Gibbs, 2015). Previous studies, both these co-authors and others, also reached the same conclusion, where activation of β3-AR, but not β2, reduced Aβ and its production and prevented the associated memory loss in an AD model (Gibbs et al., 2010; Tournissac et al., 2021); these facts support our findings. The failure of β2-AR agonists to mediate similar effects may be owed to the binding of β2-AR to Aβ leading to its dysfunction and degeneration (Wang et al., 2011), a finding that can explain the effect of the β2-AR blocker carvedilol. This notion was reviewed earlier in 1999 when it was found that carvedilol was able to bind Aβ and prevent it from forming oligomeric fibrils (Howlett et al., 1999).

The tested drugs also exerted an anti-inflammatory effect by inhibiting the IAA-mediated increase of the transcription factor NF-κB, the inflammatory surrogate TNF-α, and their upstream molecule GSK-3β in addition to the oxidative stress marker MDA; this offers another mechanism for their anti-colitic effect since inflammation plays a pivotal role in the pathomechanism of ulcerative colitis (Uddin et al., 2013; Kwiecien et al., 2014). The anti-inflammatory effect of these drugs gives another explanation for the inhibited PS1/BACE-1/Aβ axis where the inflammatory cascade was recounted to activate the PS1/BACE-1/Aβ hub (Paouri et al., 2017) and to increase the formation of ROS, which in turn elevates also the AD-related markers (Kummer and Heneka, 2008; He et al., 2017). In line with the present work, GSK-3β was reported to be activated in ulcerative colitis, where it positively regulated NF-κB (Uddin et al., 2013), which then controls pro-inflammatory mediators like TNF-α (Demarchi et al., 2003). Moreover, the inhibition of GSK-3β in an AD model robustly decreased the oligomeric Aβ load in the mouse brain (DaRocha-Souto et al., 2012) and its activation augments PS1 to enhance the formation of Aβ42 (Zhang et al., 2020).

Besides the above mechanisms, the AR modulators tested herein succeeded to augment PPAR-γ, another transcription factor known for its anti-colitic mechanism. The correlation between β3-AR and PPAR-γ has been highlighted previously by several models, but not in ulcerative colitis. Previously, the activation of the β3-AR, in a PPAR-γ-dependent manner, was able to ameliorate liver steatosis and inflammation in nonalcoholic fatty liver disease (Wang et al., 2020), to cause the browning of white adipocytes to cure obesity (Fan et al., 2019), to offer anti-atherosclerotic effects (Shi et al., 2014), and to hinder preterm labor onset (Hadi et al., 2017). Moreover, the inverse correlation between PPAR-γ and NF-κB can explain its anti-inflammatory character and its inhibition in the present ulcerative colitis model can partake in the exaggerated inflammatory cascade as reported earlier (Latella et al., 2014; Noori et al., 2020). Indeed, the link between PPAR-γ and the PS1/BACE-1/Aβ trajectory has been highlighted in different AD models, where the increased PPAR-γ decreases Aβ (Camacho et al., 2004) and silences BACE-1 (Kummer and Heneka, 2008). Moreover, the PPAR-γ agonist 15d-PGJ2 was able to inhibit the expression of PS1 in a PPARγ-dependent manner in a model of AD (Lu et al., 2018). Moreover, this receptor inhibits inflammation (Viladomiu et al., 2013) and fibrosis (Vetuschi et al., 2018) to represent a hinge upon which the tested drugs mediate partly their effects.

Our study also showed that the treatment regimens succeeded in suppressing the NOTCH1/NICD/HES-1 trajectory that was activated in the colitic group. Although several studies have reported the activation of this pathway in ulcerative colitis models (Zheng et al., 2011; Ghorbaninejad et al., 2019; Christopoulos et al., 2021), scarce data exists that can link ARs with this signaling pathway. A previous study noted that stimulation of β2-AR receptor activates NOTCH1, a fact that can explicate the lowering effect of the β-blocker carvedilol to suppress the catecholamine-induced NOTCH1 activation (Chen et al., 2014). Since to date, no data is reporting the effect of β-3 AR or mirabegron on the NOTCH pathway, one can postulate that activation of PPAR-γ along with the inhibition of the inflammatory parameters and the PS1/BACE-1/Aβ hub can be the tether. In this regard, the increased PS1 was reported to activate the NOTCH trajectory (Selkoe, 2001) and to cleave NOTCH1 to its active form and accordingly HES1(Soriano et al., 2001; Lu et al., 2018; Jfri et al., 2019). Mutually, the activation of NOTCH1 enhances Aβ (Ahn and June, 2007) and BACE-1 (Leal et al., 2012). In addition, the interplay between inflammation and the NOTCH signaling has been amply reviewed (Quillard and Charreau, 2013; Fazio and Ricciardiello, 2016) and it has been reported that NOTCH1 and HES1 in co-operation with TNF-α can suppress PPAR-γ (Maniati et al., 2011) to play a crucial role in the pathogenesis of ulcerative colitis (Ghorbaninejad et al., 2019). On the other hand, another study stated that stimulated PPAR inhibits NOTCH1 (Liu et al., 2022). These data, hence, prove the crosstalk between the assessed parameters to pin down the anti-colitic effect of the tested drugs.

Furthermore, the ability of the used drugs to shut down the NOTCH1 trajectory explains the increased GCs, where previous data showed that hypo-activated NOTCH/HES1 enhances the formation of GCs (Crosnier et al., 2006; Zheng et al., 2011), which could give a novel insight into the present anti-ulcerogenic effect of both mirabegron and carvedilol. The tested drugs were also able to preserve cell integrity by inhibiting the protein expression of *p* ^(Y654)^-β-catenin to maintain its link to E-cadherin, since the phosphorylation of β-catenin at this site interrupts the E-cadherin/β-catenin complex and cell adhesion in turn (Behrens et al., 1993; Taddei et al., 2002). The role of carvedilol to uphold the E-cadherin/β-catenin hub has been reported earlier in a liver fibrosis model (El-Wakeel et al., 2018). Moreover, the aptitude of mirabegron and carvedilol to reduce PS1 and the inflammatory markers facilitate the stabilization of the E-cadherin/β-catenin complex, since each of them can interrupt this adhesion (Nelson and Nusse, 2004; Twomey and McCarthy, 2006; Saito et al., 2014); however, the augmentation of PPAR-γ cannot be ruled out (Bocca et al., 2007).

The final mechanism by which the tested drugs alleviate IAA-induced ulcerative colitis was by their anti-fibrotic effect manifested by the reduction of the TGF-β1/α-SMA axis and the enhancement of SMAD-7, effects that were reverted by the ulcerogenic inducer. Indeed, TGF-β1 has a controversial role in the pathogenesis of ulcerative colitis (Feagins, 2010; Ihara et al., 2017), yet it was here part of the pathomechanism. The anti-fibrotic potential of the tested drugs has been reported earlier in non-ulcerative colitis models; first, carvedilol was able to decrease TGF-β1/α-SMA and increase SMAD-7 in renal (Wong et al., 2001) and hepatic (El-Wakeel et al., 2018) injury models, whereas mirabegron proved its anti-fibrotic potential against doxorubicin-induced cardiotoxicity *via* the inhibition of TGF-β/SMAD2/3 pathway (Freiwan et al., 2022). However, the aptitude of mirabegron to release noradrenaline (Mo et al., 2017) may also be one possible reason behind the augmentation of SMAD-7 (Kanamaru et al., 2001).

The inhibition of the fibrotic marker also participates in the anti-oxidant and anti-inflammatory potentials of the tested drugs, since TGF-β1 triggers redox imbalance, which in turn heightens NF-κB (Morry et al., 2017), to increase TGF-β1 (Suryavanshi and Kulkarni, 2017) in a feedforward pattern. Moreover, the stimulation of PPAR-γ increases SMAD7 to inhibit the TGF-β1 signaling pathway (Vallée et al., 2017), an effect that can be linked to its anti-oxidant character (Morel and Singer, 2014; Morry et al., 2017). In addition, TGF-β1 has been reported to crosstalk with PS1 in normal and pathological conditions (Ren et al., 1999), with GSK-3β (Baarsma et al., 2011), and with NOTCH signaling (Yi et al., 2017). Taken together*,* the current study sheds more light on the possible anti-colitic mechanisms of mirabegron/carvedilol by turning off the injurious effect of these trails.

## Conclusion

Here we found that exogenous administration of mirabegron and/or carvedilol caused an upturn in the colon structure and GCs count in a model of ulcerative colitis. This anti-colitic effect may be attributed to the inhibition of oxidative stress, inflammation, fibrosis, and barrier dysfunction. These effects were partly achieved by inhibiting several harmful intersecting signaling pathways; *viz*., PS1/BACE-1/Aβ, NOTCH1/NICD/HES1, GSK-3β/NF-κB/TNF-α, MDA, and TGF-β1/α-SMA, and enhancing PPAR-γ, SMAD-7, as well as cell integrity (E-cadherin/β-catenin axis).

## Data Availability

The original contributions presented in the study are included in the article/Supplementary Material, further inquiries can be directed to the corresponding author.

## Abbreviations

Aβ, amyloid beta; AJ, adherens junction; APP, amyloid precursor protein; BACE-1, beta-site APP cleaving enzyme-1; CR, carvedilol; GSK-3β, glycogen synthase kinase-3 beta; HES1, hairy and enhancer of split-1; MA, mirabegron; MDA, malondialdehyde; MTC, Masson’s Trichrome for collagen fibers; NF-κB, nuclear factor-kappa B; NICD, notch intracellular domain; PPAR-γ, peroxisome proliferator-activated receptor gamma; PS1, presenilin; α-SMA, α-smooth muscle actin; TGF-β1, transforming growth factor-beta 1; TNF-α, tumor necrosis factor-alpha.
