# Supplementary material for: The novel anti-colitic effect of β-adrenergic receptors via modulation of PS1/BACE-1/Aβ axis and NOTCH signaling in an ulcerative colitis model
Source: Front Pharmacol. 2022 Oct 25;13:1008085. doi: 10.3389/fphar.2022.1008085 (PMC9641009; doi:10.3389/fphar.2022.1008085)

Supplementary Data

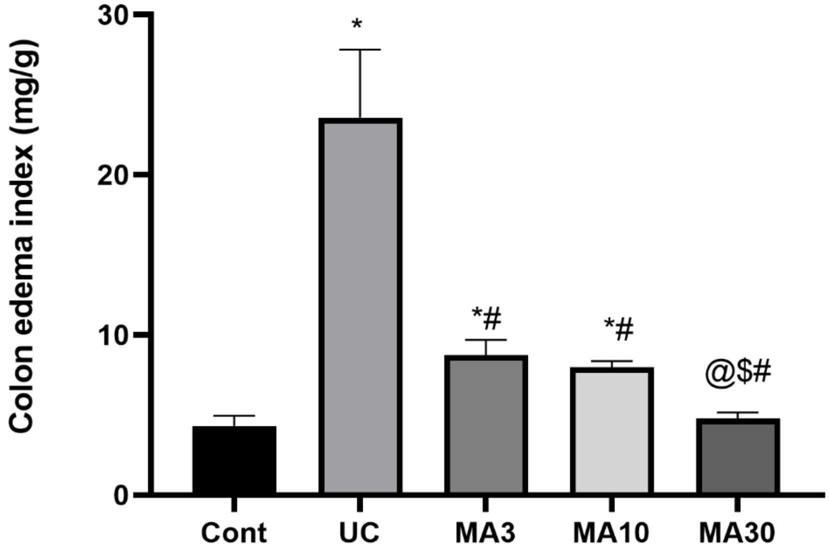

Figure 1S: Effect of MA3, MA10 and MA30 on colon edema index in rats with IAA-induced UC. Data are presented as mean ± SD (n = 6). Statistical analysis was carried out using one-way ANOVA followed by Tukey's *posthoc* test; $P < 0.05$. As compared to (*) CONT, (#) UC, (@) MA3 and ($) MA10 groups. CONT: control; MA: mirabegron and UC: ulcerative colitis.

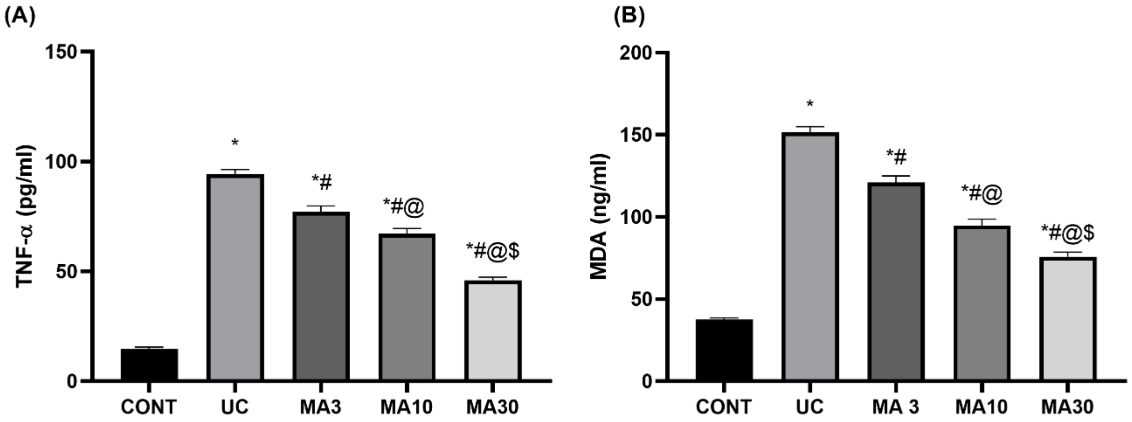

Figure 2S: Effect of MA3, MA10, and MA30 on the colon protein content of (A) TNF-α and (B) MDA in rats with IAA-induced UC. Data are presented as mean ± SD (n = 6). Statistical analysis was carried out using one-way ANOVA followed by Tukey's *post-hoc* test; $P < 0.05$. As compared to (*) CONT, (#) UC, (@) MA3, and ($) MA10 groups. CONT: control; MA: mirabegron; MDA: malondialdehyde; TNF-α: tumor necrosis factor-alpha; UC: ulcerative colitis.

<u>Western uncropped photos</u>

BACE-1

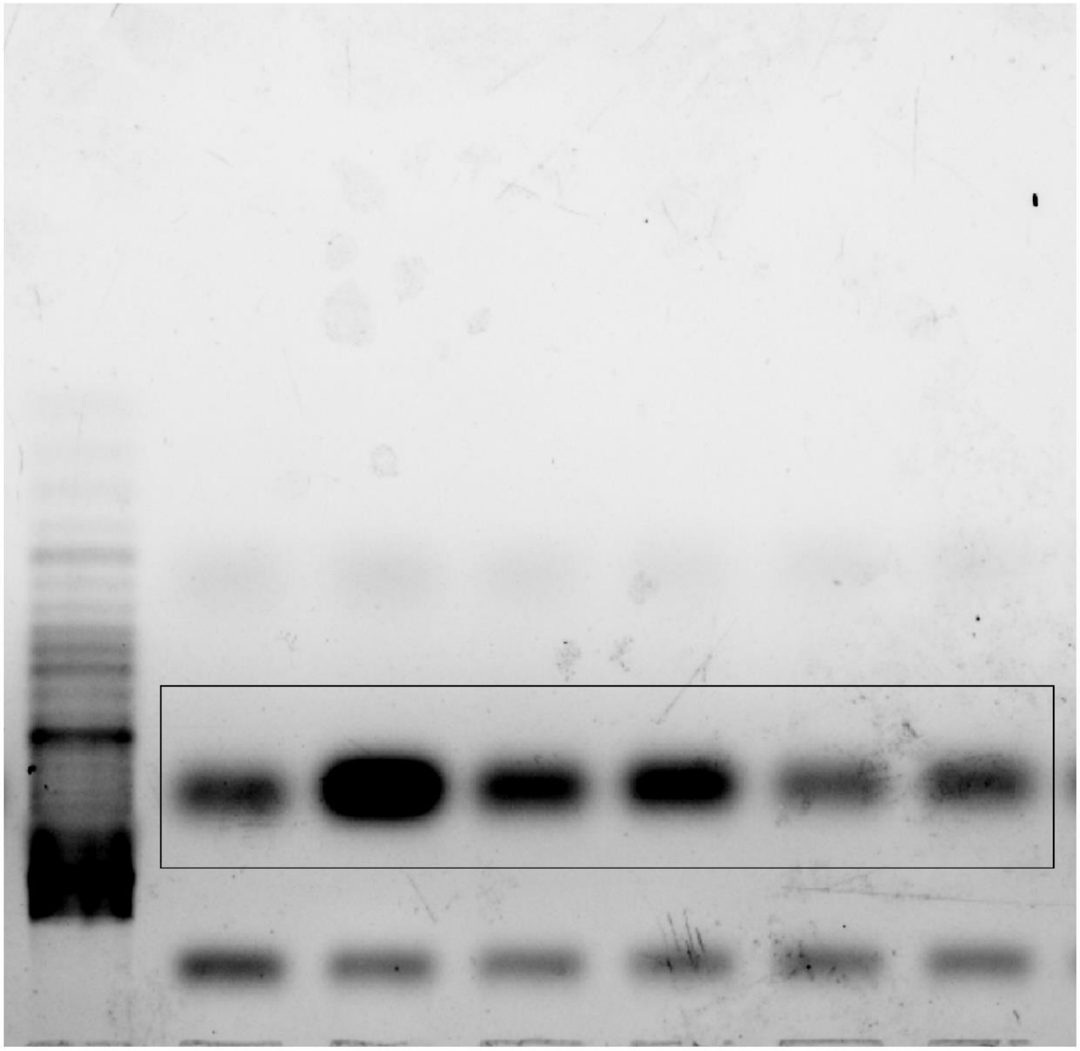

***p*Y654-β-Catenin**

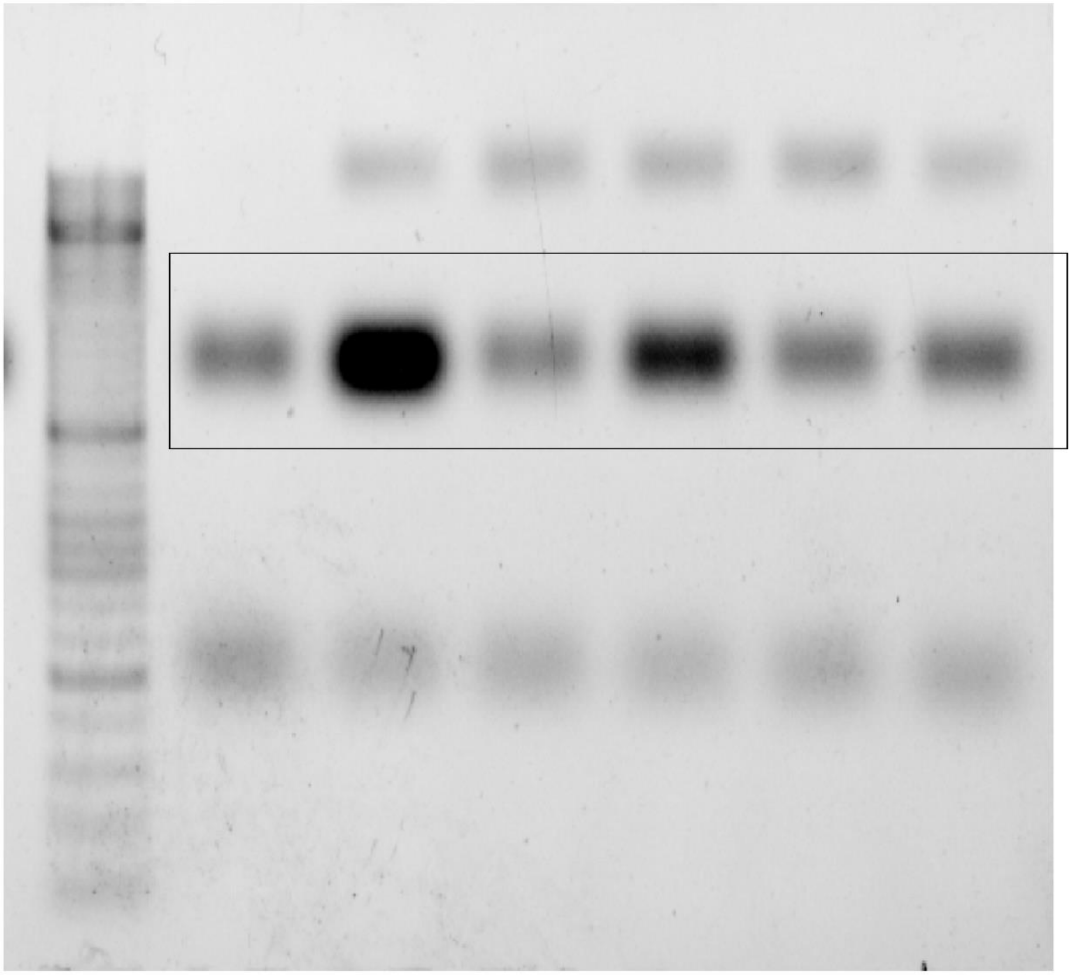

pS9-GSK3-β

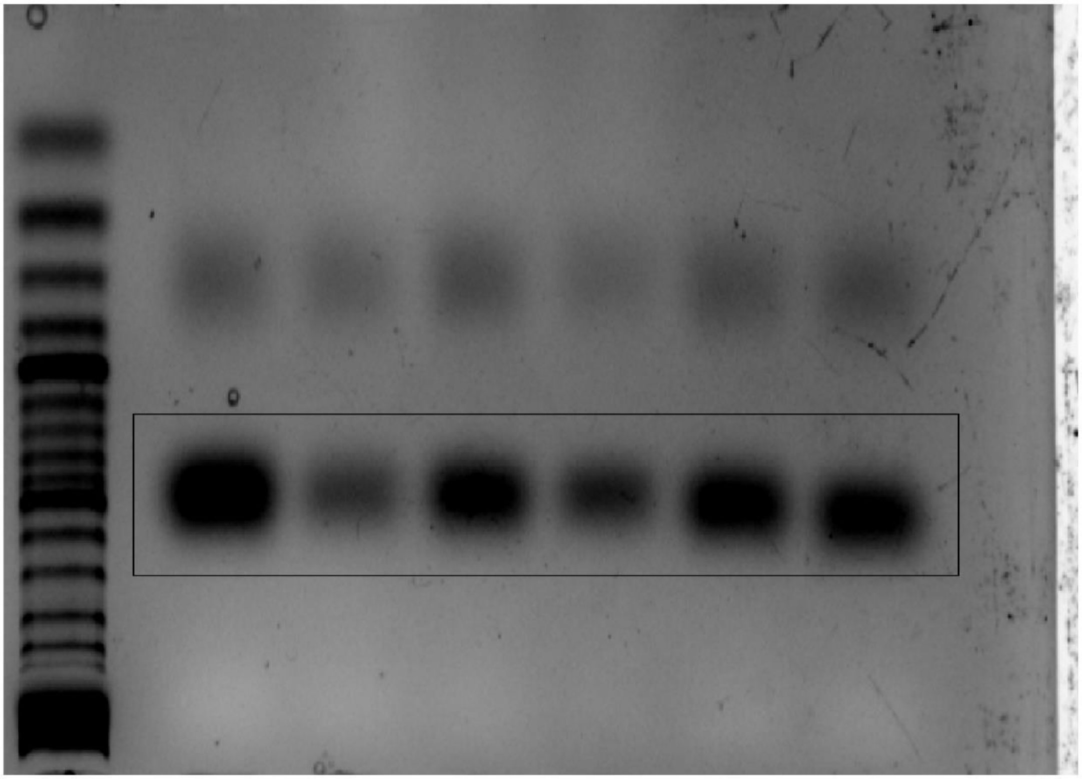

NICD

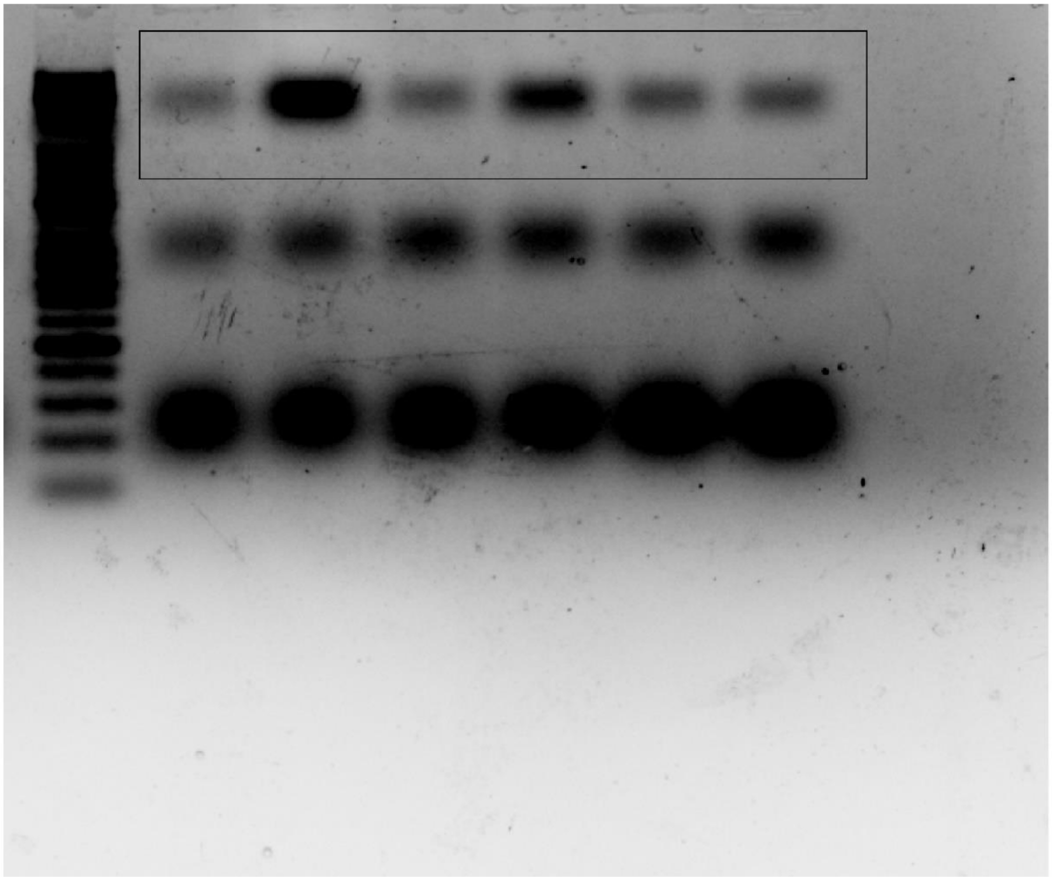

***p*S353-Presenilin**

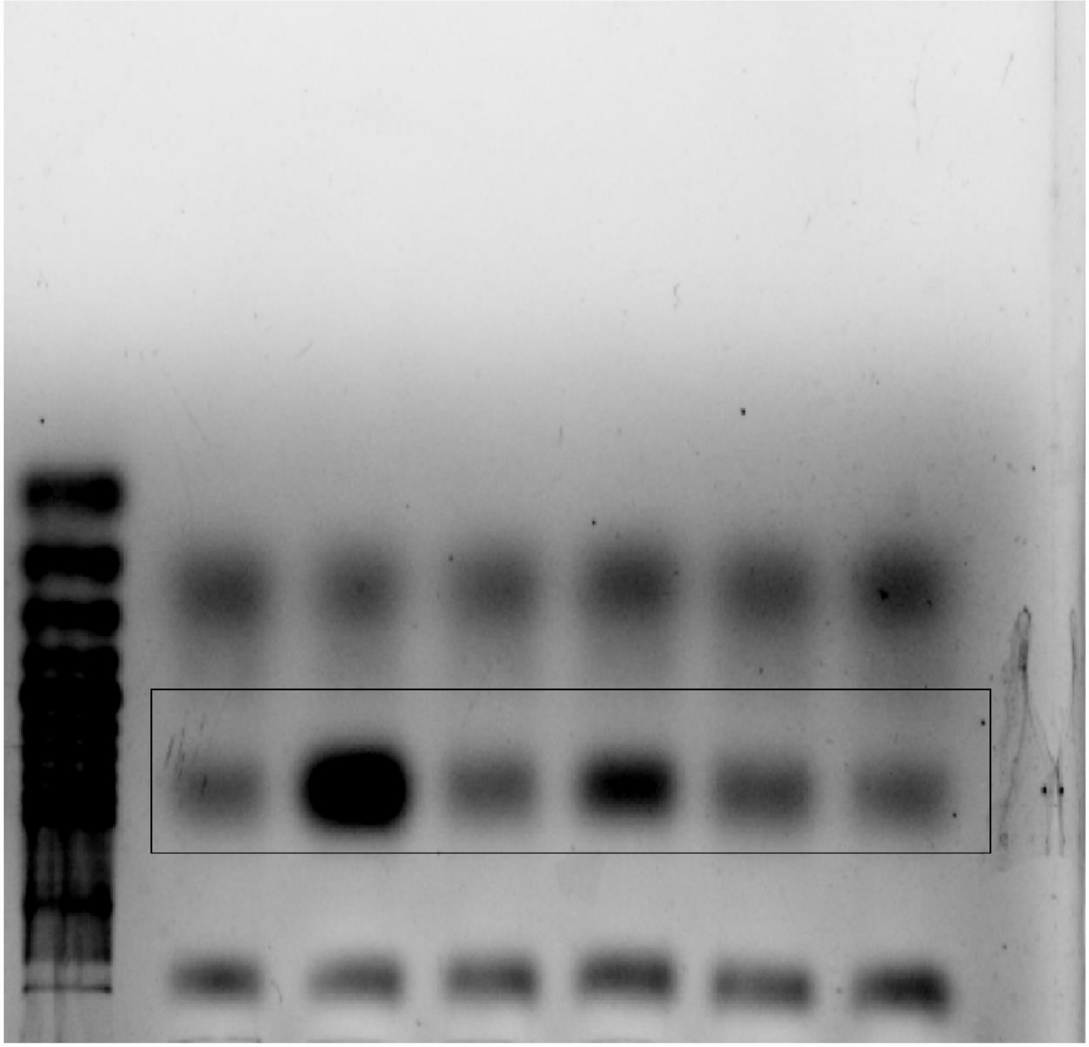

Total presenilin

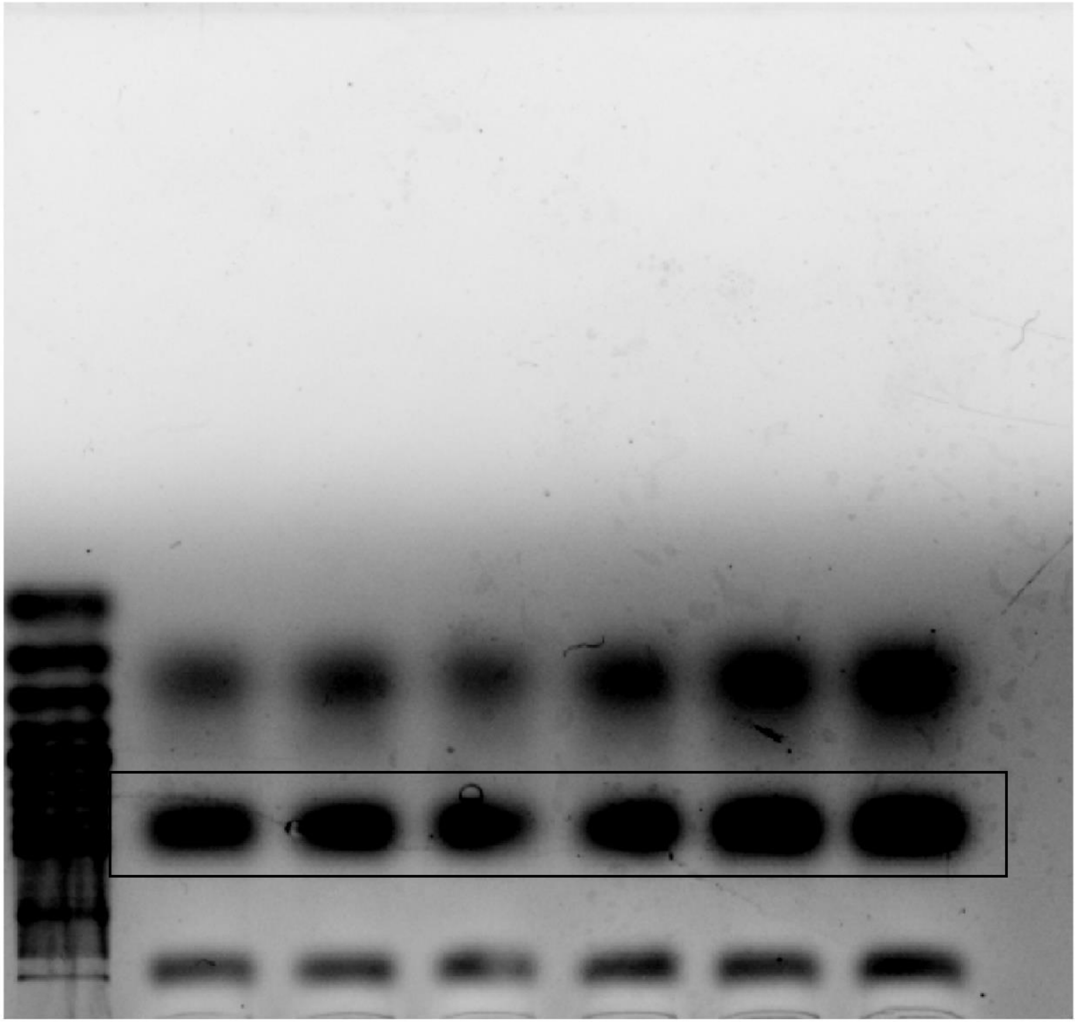

β-Actin

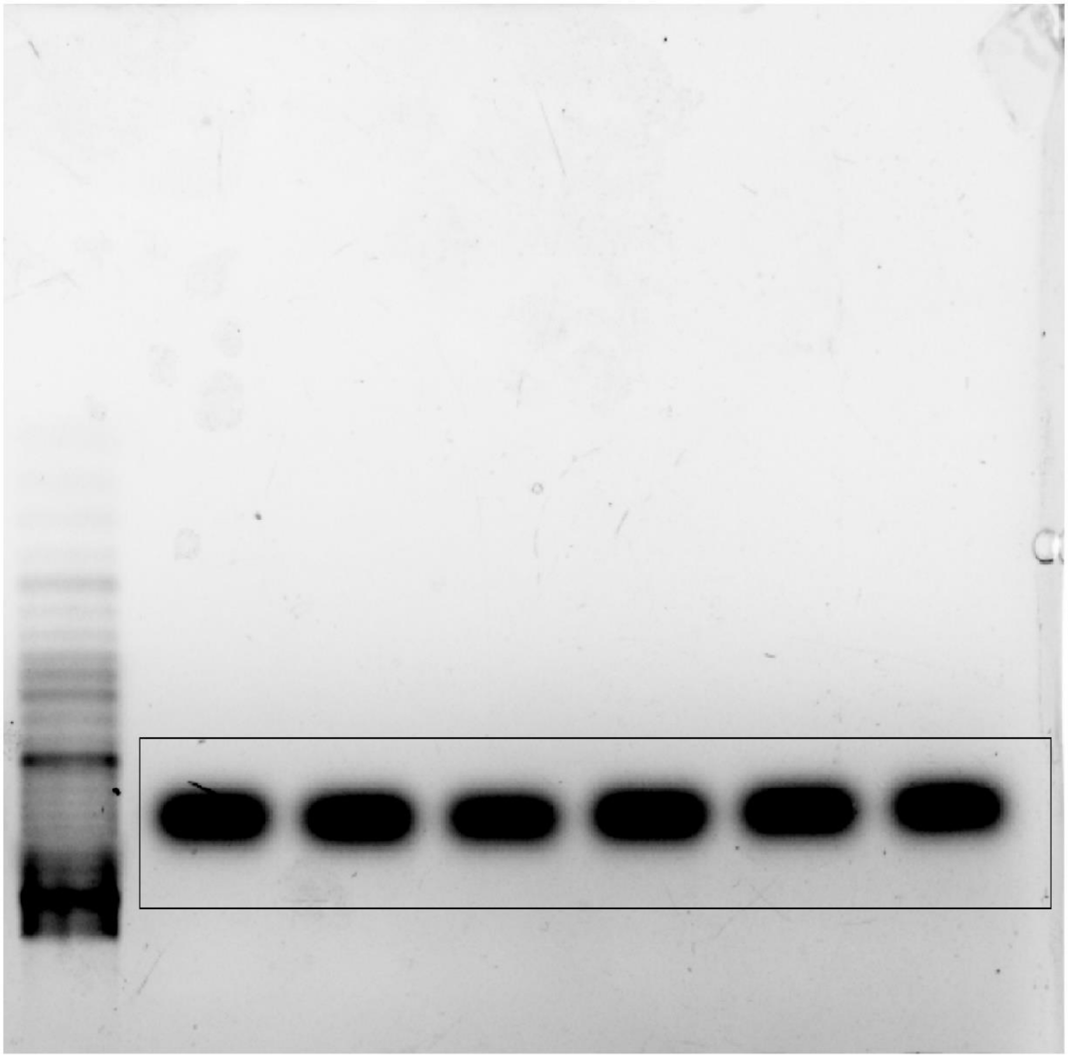

Supplement: Supplementary file 1 [file DataSheet1.pdf]
